# Patient-specific versus Organisational Barriers to Program Adherence: A Multivariate Analysis

**DOI:** 10.5334/ijic.4212

**Published:** 2019-03-15

**Authors:** Sara Fokdal Lehn, Ann-Dorthe Zwisler, Solvejg Gram Henneberg Pedersen, Thomas Gjørup, Lau Caspar Thygesen

**Affiliations:** 1 Department of Medicine, Holbæk University Hospital, Smedelundsgade 60, Holbæk, DK; 2 Knowledge Centre for Rehabilitation and Palliative Care, Odense University hospital and University of Southern Denmark, Vestergade 17, Nyborg, DK; 3 Danish Knowledge Centre for Rehabilitation and Palliative Care, University of Southern Denmark and Odense University hospital, Vestergade 17, Nyborg, DK; 4 Department of Medicine, Geriatric section, Holbæk University Hospital, Smedelundsgade 60, Holbæk, DK; 5 Emergency Clinic, Gentofte Hospital, Kildegårdsvej 28, Hellerup, DK; 6 National Institute of Public Health, University of Southern Denmark, Studiestræde 6, København, DK

**Keywords:** implementation, adherence, integrated care program, older patients, organizational factors, patient-specific factors

## Abstract

**Introduction:**

This article explores the influence of patient-specific and organisational factors on adherence to program guidelines in an integrated care program targeting older patients.

**Methods:**

The integrated care program aimed to offer post-discharge follow-up visits by a municipality nurse and the general practitioner to frail older patients after discharge from hospital. Adherence was measured as *step 1*) successful referral from the hospital and *step 2*) completed post-discharge follow-up visit. We followed a cohort of 1,659 patients who were selected to receive a post-discharge follow-up visit in 2014. We obtained unique data from hospitals, municipalities and from administrative registers.

**Results:**

We found substantial lack of adherence in both steps of the program: 69% adherence in step 1 and 54% adherence in step 2. In step 1, adherence was related to hospital, and receiving municipal home care prior to admission. In step 2, level of adherence varied according to municipality, the type of general practitioner and the patient’s gender.

**Conclusion:**

We found that adherence was strongly related to organisational factors. Adherence differed significantly at all organisational levels (hospital, municipality, general practice), thus indicating challenges in the vertical integration of care. Gender influenced adherence as the only patient-related factor.

## Introduction

Integrated care aims to enhance connectivity of the cure and care for patients with care needs that cut across multiple services, providers and settings [1]. However, integrated care programs face various challenges in the existing health care system, which in turn impedes the expected patient outcomes [2,3]. The concept of implementation refer to an actively planned and deliberately initiated effort to bring a given object into action [4]. One way to measure the level of implementation of a new integrated care practice is by observing the level of adherence to the planned procedures [2]. ‘Program adherence’ refer to coverage, frequency, duration and content, of an intended intervention; hence, the degree to which ‘a program service or intervention is being delivered as it was designed or written’ [2]. Moreover, we need more knowledge about the factors that are affect adherence or non-adherence within integrated care programs. Enablers of implementation of integrated care programs include context, such as leadership that fostered shared vision of integrated care, and the flexibility of a program to align with the need of the local population [3]. Still, new initiatives of integrated care should be diffused equally across target groups, and should not differ between specific subgroups, e.g. socio-economic groups [5]. Nonetheless, one study showed that adherence to a relatively simple, short-term alcohol prevention program in primary care was influenced by non-clinical factors related to the patient or the organisation; i.e. gender, occupation, education and the type of general practice clinics [6]. In addition, when turning to initiatives addressing older patients, the multifaceted health problems designed programs are typically more complex, involving multiple stakeholders such as general practice, home care, outpatient clinics; such programs often face acute challenges of implementation across health care organisations [7,8]. There is lack of knowledge about factors at the level of patients and provider organisations that can facilitate or impede the implementation of integrated care programs [7,9]. Hence, more knowledge about the factors associated with adherence and how they interact can aid researchers and clinicians in refining their interventions and preventing systematic differences in program coverage among targeted patient groups [6].

In the present study, we focus on adherence to a nation-wide integrated care program that aims to prevent readmission and suboptimal continuity of care for older medical patients. The core of the program consists of joint home visits by a municipality nurse and a general practitioner following hospital discharge. In a randomized control trial, the program had been shown to reduce readmission risk [10]. In Denmark, the program was made mandatory on a national level in 2013 [11]. As an application to the existing health care system, the program entailed new types of patient contact for both the municipal nurse and general practitioners, since not all referred patients received municipal nursing or home care service on a regular basis, and since the general practitioner consultation was now taken outside the clinical setting. Understanding the factors affecting adherence to this new form of integrated care is important for successful implementation in other inter-organisational programs, and we hypothesis that both patient-specific and organisational factors influence program adherence.

This article investigates the degree to which adherence to this integrated care program targeting older patients is associated with patient-specific factors, i.e. demographic, social and health-related factors, and with organisational factors. Thus, we explore the association between relevant factors and indicators of adherence in the post-discharge follow-up program.

## Method

We conducted an observational study of patients who were consecutively screened during hospital admission and found eligible for post-discharge follow-up visits in Region Zealand, Denmark, in 2014.

### Setting

The Danish health care system is an open-access, tax-funded system. General practitioners serve as gatekeepers to specialized health care. General practitioners are independent operators who enter into contracts with the regional health authorities [12]. Municipalities provide practical and nursing assistance to elderly patients and to people with functional disabilities. A municipality nurse is a registered nurse employed by the municipality who may either coordinate the home visit or carry it out. In the secondary health sector, hospitals are responsible for most of the specialized treatment [13].

### The integrated care program

We studied adherence to the integrated care initiative, post-discharge follow-up program. Municipal nurses and the general practitioners performed joint visits in the patient’s home within seven days from discharge. During the post-discharge follow-up visit, the general practitioner and the municipal nurse reviewed the treatment plan and medication with the patient. Completion of the post-discharge follow-up visits depended on a chain of procedures, starting at the hospital, where patients were screened to be eligible for the program. Patients aged 78 years and older were systematically screened, although younger patients could be included. Both nurses and physicians participated in the screening, after which nurses were responsible for referral to the municipal care staff. Referral was transmitted through a digital communication form in a secured system. Municipal nurses coordinated visits with the patient’s general practitioner and informed patients about when the visit would take place.

### Study cohort

In 2014, data was systematically and extraordinarily recorded for the purpose of program monitoring at hospitals and municipalities in Region Zealand, Denmark. This study is based on a cohort of total 1,659 patients who were enrolled in the post-discharge follow-up program at six acute hospitals in Region Zealand, Denmark. For eligibility criteria please see Table 1. The analysis described below is based on both this total cohort (step 1), and a nested cohort of 956 patients who were successfully referred to the municipality (step 2).

**Table 1:** Eligibility criteria for the post-discharge follow-up program, Region Zealand, 2014. Patients who were not discharged to an intermediate care facility, with at least 3 of the below listed readmission risk factors, who were additionally clinically assessed to be eligible, were referred to the program.

Suspected cognitive disturbance or problems
Substance abuse that influences functional level
Psychiatric disease that influences functional level
Disadvantaged social network
Large loss in ability to carry out the activities of daily living
Malnutrition
Severe chronic, progressive, somatic or psychiatric disease
Six or more prescription drugs
High need for coordination of treatment and care
Acute hospital contact within the previous 6 month
Inconvenient residential facilities
Increased municipal services
The patient was not in contact with municipal services
History of fall
Clinical assessment (mandatory)

### Measures of adherence

**Figure 1:**
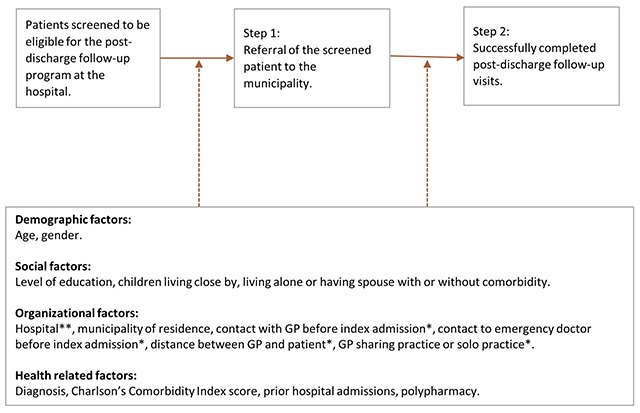
Adherence measures related to the post-discharge follow-up program and potential factors affecting adherence. * Variables not analysed in relation to adherence step 1. ** Variables not analysed in step 2.

Besides hospital data for the enrolled patients, we obtained municipal data regarding received referrals for the post-discharge follow-up program and registration of completed post-discharge follow-up visits. Referral from hospital to municipality setting, and the actual post-discharge follow-up visits with the presence of general practitioner and municipal nurse were essential to the post-discharge follow-up program. Hence, adherence is categorized by the two distinct steps in the post-discharge follow-up program (Figure 1):

Step 1: Referral of the screened patient to the municipality.

Step 2: Successfully completed post-discharge follow-up visits.

Successfully completed post-discharge follow-up visits can be defined as joined visits by the general practitioner and municipality nurse within one month from discharge.

### Variables potentially associated with adherence

Monitoring data from hospitals and municipalities, as well as information from Danish registers, provided individualized data on several aspects of demographic, social, organisational and health conditions [14]. Analysis of step 1 included 11 variables, whereas analysis of step 2 included 15 variables, since analysis of step 1 did not include variables related to general practitioner or emergency doctor.

#### Health-related variables

Information about admissions, diagnosis and length of stay was obtained from the Danish National Patient Register [15], and information about the patient’s use of medication was obtained from the Danish National Prescription Registry [16]. The main diagnosis was categorized into *atypical symptoms* and *other diagnosis*. Atypical symptoms included diagnoses that did not relate to a specific disease, i.e. ICD-10 groups R (‘Symptoms, signs and abnormal clinical and laboratory findings, not elsewhere classified’) and Z (‘Factors influencing health status and contact with health services’). The Charlson comorbidity score was calculated using information about primary and secondary diagnoses from all hospital contacts up to 10 years before index admission [17]. Polypharmacy was defined as the use of five or more different prescribed drugs within a period of three months prior to admission.

#### Demographic variables

Age and gender were obtained from the Civil Registration System [18]. Statistics Denmark provided information on city size based on patients’ addresses.

#### Social variables

Marital status was obtained from the Civil RegistrationSystem [18]. Educational level was obtained from the Danish Education Register [19], income data from the Income Statistics Register [20], and information about children from the Fertility Database and the Adoption Register [21].

#### Organisational variables

Data on patients’ contact with general practitioners and emergency medical service, type of general practitioner was obtained from the National Health Service Register [22]. Information on municipal basic home services was obtained from municipality registrations. Distance between the patient’s home and the general practitioner’s clinic was calculated by Statistics Denmark using the general practitioner clinic address obtained from the National Health Service Register and the patients’ address from the Civil Registration System. The precise distance was calculated using the Danish geographical information system.

### Analysis

We performed both univariate logistic regression analysis, adjusted only for age and gender, and we performed multiple logistic regression analysis, including all variables for both adherence measures. The first analytical model refers to the odds that the municipality received a referral when patients had been screened as eligible for the post-discharge follow-up program (Figure 1, adherence step 1). The second analytical model refers to the odds that patients receive post-discharge follow-up visits when they have been referred (Figure 1, adherence step 2).

#### Missing values

We expected a low level of missing observations due to the high coverage of Danish registers [14]. However, missing values appeared in relation to the ‘highest level of education’ variable (7% missing values), type of general practitioner (9% missing values), and distance to general practitioner (21% missing values). For individuals born in Denmark in 1945 or later, the registration of highest level of education is 97% complete. Nevertheless, the proportion of missing information about educational level increases with decreasing birth cohorts for individuals born before 1945 [19]. Taking higher education was not common in Denmark before 1945 [23]. Hence, in this study, we impute missing values of education using the lowest categorized level of education ‘Primary school’. Missing values appear in relation to the general practitioner, since service numbers and practice addresses change over time. Since the proportion of missing values for type of general practitioner is below 10%, we chose to retain this variable and exclude those cases with missing values from the analysis. However, in the case of distance between general practitioner and the patient’s home, the proportion of missing values is relatively high (21%); hence, we chose to exclude this variable in the multiple analysis.

## Results

**Figure 2:**
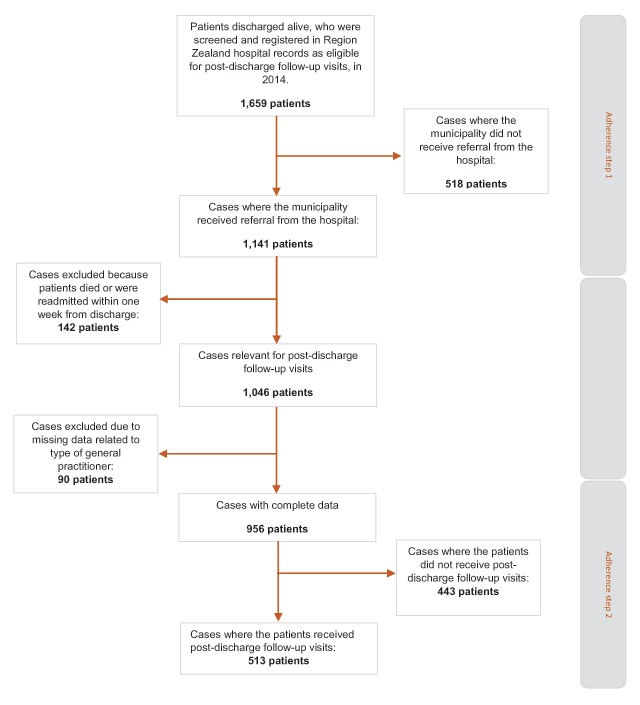
Patient flowchart, describing the patient cohort and different levels of analysis.

A cohort of 1,659 patients was screened and found eligible in the post-discharge follow-up program in Region Zealand in 2014 (Figure 2). Of these, the municipalities received referrals for 1,141 patients, which is just 69% of the patient cohort. A group of patients (142 patients) died or were readmitted within seven days of discharge and were thus unable to receive post-discharge follow-up visits within this predefined period. Hence, 1,046 patients were eligible to receive post-discharge follow-up visits. However, 90 cases were excluded from the analysis due to missing values of type of general practitioner. Of the 956 patients included in the analysis of adherence in step 2, and for whom the municipality staff received referral, 513 patients, which is just 54% of the patient group, received the post-discharge follow-up visit (Figure 2).

**Figure 3:**
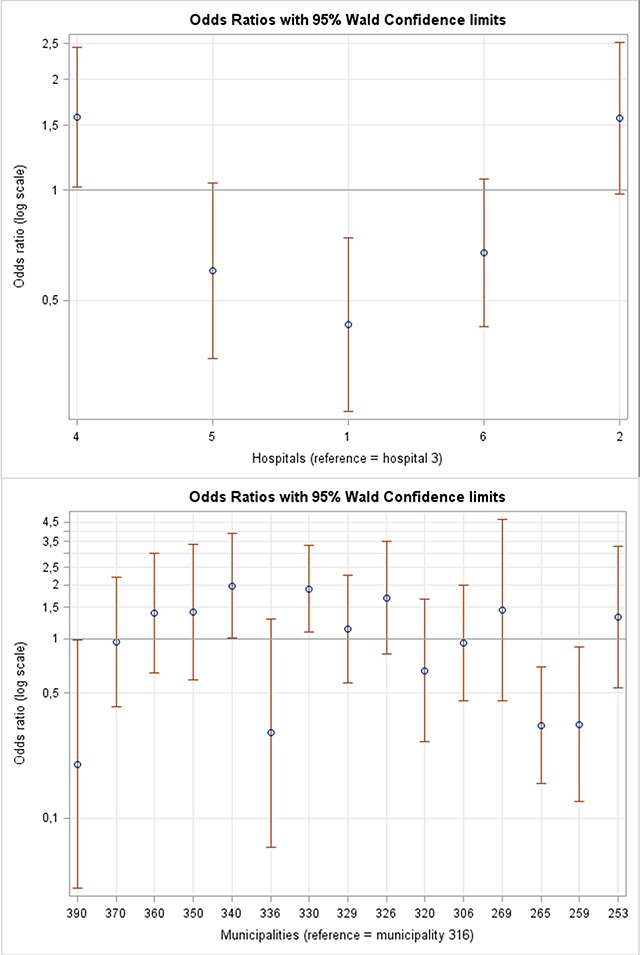
Organisational factors. In the top; adjusted odds ratio plots with 95% confidence intervals for successful referral from hospital to municipality (step 1, full model) stratified by hospitals and for receiving post-discharge follow-up visits. Below; adjusted odds ratio plots with 95% confidence intervals for successfully completed post-discharge follow-up visits (step 2, full model) stratified by municipality. Note that municipality no. 372 is not represented in this figure due to the low number of observations (6 observations) and very broad 95% confidence interval.

Analysis of adherence step 1 was based on the total cohort of 1,659 patients who were screened at the hospital and found eligible for post-discharge follow-up patients (Table 2). It was mainly organisational factors, which influenced whether the municipality received referral when patients were found eligible. Thus, both hospital settings (Figure 3) and whether patients received homecare prior to discharge were associated with whether municipality received referral both in the age- and gender-adjusted models and in the full regression model. In the full logistic regression model, two out of five hospitals showed significantly lower odds ratio of receiving referral in the municipality, ranging from 0.27 (CI 0.18–0.42) to 0.63 (CI 0.41–0.98), compared to the reference hospital, Hospital 3 (south). In addition, multiple logistic regression showed slightly higher odds ratio of referral if the patients received homecare prior to index discharge (OR 1.37 (CI 1.10–1.70)), compared to patients who did not. Age- and gender-adjusted analysis showed that if the patients’ main diagnosis indicated atypical symptoms, the odds of referral (OR 1.28 (CI 1.01–1.62)) was higher compared to other main diagnosis. However, this difference was insignificant in the multiple regression model.

**Table 2:** Analysis of adherence step 1; factors related to referral from hospital to municipal setting. The middle columns show results from logistic regression model adjusted for age and gender, and the right hand columns show results of the full logistic regression model (n = 1659 patients).

Factor	Value	Absolute numbers (%)	Each variable adjusted for age and gender		Full model analysis	

			OR (CI)	P-value	OR (CI)	P-value

Demographic						

Age	40–64 years	59 (4)	0.70 (0.40–1.20)	0.54	0.66 (0.38–1.17)	0.42
median = 84 years	65–77 years	369 (22)	0.98 (0.75–1.27)		1.03 (0.79–1.35)	
	78–89 years	960 (58)	1		1	
	90–102 years	271 (16)	1.08 (0.80–1.45)		0.87 (0.63–1.20)	
Gender	Male	676 (41)	1	0.69	1	0.56
	Female	983 (59)	0.96 (0.77–1.19)		0.93 (0.73–1.18)	
**Social**						

Education	Higher education	190 (12)	1	0.90	1	0.71
	Vocational education	474 (29)	0.92 (0.64–1.33)		0.87 (0.59–1.27)	
	Primary school	889 (54)	0.96 (0.68–1.35)		0.87 (0.61–1.23)	
		*Missing: 106 (6)*				
Children living close by	No	633 (42)	1	0.79	1	0.87
	Yes	884 (58)	1.03 (0.83–1.28)		1.02 (0.82–1.28)	
		*Missing: 2 (0)*				
Spouse with or without comorbidity	Spouse with CCI* of 0–1	408 (25)	1	0.57	1	0.69
	Spouse with CCI* of 2+	150 (9)	0.96 (0.65–1.44)		0.92 (0.61–1.39)	
	No spouse	1101 (66)	1.12 (0.86–1.46)		1.08 (0.82–1.42)	
**Organisational**						

Hospital	Hospital 1 (south-west)	110 (7)	**0.42 (0.25–0.72)**	**<0.0001**	**0.43 (0.25–0.74)**	**<0.0001**
	Hospital 5 (north-east)	117 (7)	**0.54 (0.32–0.91)**		0.60 (0.35–1.05)	
	Hospital 6 (mid-east)	326 (20)	**0.61 (0.39–0.95)**		0.68 (0.43–1.07)	
	Hospital 3 (south)	122 (7)	1		**1**	
	Hospital 2 (north-west)	326 (20)	1.46 (0.92–2.31)		1.57 (0.97–2.52)	
	Hospital 4 (mid-west)	658 (40)	1.50 (0.98–2.29)		**1.58 (1.02–2.45)**	
Received municipal homecare prior to admission?	No	770 (46)	1	**0.0002**	1	**0.01**
	Yes	889 (54)	**1.50 (1.21–1.86)**		**1.37 (1.10–1.70)**	
**Health related**						

Main diagnosis	Other diagnosis	1199 (72)	1	**0.04**	1	0.13
	Atypical symptoms	460 (28)	**1.28 (1.01–1.62)**		1.22 (0.95–1.53)	
Charlson comorbidity index score	0–1	663 (40)	1	0.16	1	0.08
	2–3	591 (36)	1.18 (0.92–1.51)		1.19 (0.92–1.54)	
	4+	405 (24)	0.91 (0.70–1.19)		0.86 (0.64–1.56)	
Previous hospitalization within 3-month period	No	813 (49)	1	0.40	1	0.72
	Yes	846 (51)	0.91 (0.74–1.13)		0.96 (0.74–1.20)	
Polypharmacy	No	528 (32)	1	0.61	1	0.68
	Yes	1131 (68)	1.06 (0.85–1.32)		1.05 (0.83–0.83)	

* CCI = Charlson Comorbidity Index Score.

The odds of receiving post-discharge follow-up visits when patients have been referred to the program (adherence step 2) was associated with both demographic and organisational factors (Table 3); gender, municipality (Figure 3) and type of general practitioner. In the full logistic regression model, two municipalities showed higher odds of receiving post-discharge follow-up visits, with odds ratio ranging from 1.96 (CI 1.18–3.26) to 2.11 (CI 1.11–4.00), and two municipalities showed lower odds, with an odds ratio ranging from 0.20 (CI 0.05–0.77) to 0.34 (CI 0.16–0.69). Likewise, patients registered with a solo practicing general practitioner had lower odds (OR = 0.73 (CI 0.54–0.98)) of receiving post-discharge follow-up visits than patients registered with a general practitioner in a shared practice. Patients who had contact with a general practitioner within last month immediately prior to index admission had lower odds (odds ratio = 0.73 (CI 0.53–1.03)) of receiving post-discharge follow-up visits compared to those who did not, which was significant only in the age- and gender-adjusted model. This finding may reflected that GPs were less likely to visit patients with whom they were already in close contact.

**Table 3:** Analysis of adherence, step 2; factors related to odds of receiving post-discharge follow-up visits. The middle columns display results from logistic regression model adjusted for age and gender, and the right-hand columns show results of the full logistic regression model (n = 956 patients).

Factor	Value	Absolute numbers (%)	Each variable adjusted for age and gender		Full model analysis	

			OR (CI)	P-value	OR (CI)	P-value

Demographic						

Age	40–64 years	26 (3)	0.76 (0.34–1.69)	0.12	0.59 (0.25–1.42)	0.07
median = 84 years	65–77 years	213 (22)	1.38 (0.999–1.91)		1.33 (0.94–1.89)	
	78–89 years	549 (57)	1		1	
	90–102 years	168 (18)	0.89 (0.63–1.26)		0.77 (0.53–1.13)	
Gender	Male	562 (59)	1	**0.01**	1	0.01
	Female	394 (41)	**1.42 (1.09–1.84)**		**1.55 (1.14–2.10)**	
**Social**						

Education	Higher education	110 (12)	1	0.83	1	0.46
	Vocational education	271 (28)	1.07 (1.68–1.68)		1.10 (0.64–1.78)	
	Basic school	517 (54)	0.98 (0.64–1.48)		0.90 (0.58–1.41)	
		*Missing: 58 (6)*				
Children living close by	No	405 (42)	1	0.83	1	0.82
	Yes	551 (58)	1.03 (0.79–1.34)		103 (0.78–1.34)	
Spouse with or without comorbidity (based on Charlson Comorbidity Index/CCI)	Spouse with CCI* of 0–1	231 (24)	1	0.76	1	0.47
	Spouse with CCI* of 2+	83 (9)	1.12 (0.67–1.85)		1.15 (0.67–1.98)	
	No spouse	642 (67)	1.13 (0.81–1.57)		1.25 (0.88)	
**Organisational**						

Municipality	Municipality no. 390	19 (2)	**0.18 (0.05–0.66)**	**<0.0001**	**0.20 (0.05–0.77)**	**<0.0001**
	Municipality no. 265	65 (7)	**0.33 (0.16–0.66)**		**0.34 (0.16–0.69)**	
	Municipality no. 259	30 (3)	0.43 (0.18–1.06)		0.42 (0.17–1.05)	
	Municipality no. 336	19 (2)	0.59 (0.21–1.67)		0.54 (0.19–1.53)	
	Municipality no. 320	35 (4)	0.86 (0.39–1.90)		0.84 (0.37–1.88)	
	Municipality no. 306	50 (5)	0.98 (0.49–1.99)		0.94 (0.46–1.93)	
	Municipality no. 316	88 (9)	1		1	
	Municipality no. 370	40 (4)	1.02 (0.48–2.18)		0.96 (0.44–2.08)	
	Municipality no. 329	67 (7)	1.13 (059–2.15)		1.15 (0.60–2.23)	
	Municipality no. 350	31 (3)	1.31 (0.57–3.00)		1.35 (0.58–3.13)	
	Municipality no. 253	31 (3)	1.18 (0.51–2.71)		1.40 (0.59–3.32)	
	Municipality no. 360	50 (5)	1.35 (0.66–2.74)		1.43 (0.68–2.94)	
	Municipality no. 269	16 (2)	1.58 (0.52–4.79)		1.68 (0.54–5.18)	
	Municipality no. 326	78 (8)	1.84 (0.98–345)		1.89 (0.996–3.58)	
	Municipality no. 330	250 (26)	**1.89 (1.15–3.12)**		**1.96 (1.18–3.26)**	
	Municipality no. 340	81 (8)	**2.20 (1.17–4.15)**		**2.11 (1.11–4.00)**	
	Municipality no. 376	6 (1)	6.53 (0.73–58.79)		7.02 (0.75–65.80)	
Distance to general practitioner (n = 830 patients)	0–4205 m	631 (66)	1	0.30		
	4206–15000 m	169 (18)	0.85 (0.61–1.20)		na	na
	15000 + m	30 (3)	0.60 (0.28–1.27)		na	na
		*Missing: 126 (13)*				
Contact with general practitioner within one month prior to index admission	No	183 (19)	1	0.07	1	0.19
	Yes	773 (81)	0.73 (0.53–1.03)		0.78 (0.54–1.13)	
Type of general practitioner	Shared	656 (69)	1	**0.02**		**0.04**
	Solo	300 (31)	**0.71 (0.54–0.94)**		**0.73 (0.54–0.98)**	
Contact with emergency doctor within one month prior to index admission	No	607 (63)	1	0.54	1	0.58
	Yes	349 (37)	0.92 (0.71–1.20)		0.92 (0.69–1.23)	
Received municipal homecare prior to admission?	No	405 (42)	1	0.97	1	0.56
	Yes	551 (58)	1.00 (0.77–1.29)		0.92 (0.69–1.22)	
**Health related**						

Main diagnosis	Other diagnosis	662 (69)	1	0.66	1	0.85
	Atypical symptoms	294 (31)	1.06 (0.81–1.41)		0.97 (0.71–1.32)	
Charlson comorbidity index score	0–1	386 (40)	1	0.38	1	0.40
	2–3	356 (37)	1.19 (0.89–1.60)		1.23 (0.90–1.68)	
	4+	214 (22)	1.23 (0.87–1.74)		1.22 (0.83–1.79)	
Previous hospitalization within 3-month period	No	489 (51)	1	0.51	1	0.78
	Yes	467 (49)	0.92 (0.71–1.19)		0.96 (0.72–1.23)	
Polypharmacy	No	307 (32)	1	0.93	1	0.99
	Yes	649 (68)	0.99 (0.75–1.30)		1.00 (0.74–1.34)	

* CCI = Charlson Comorbidity Index Score.

## Discussion

This study has analysed a two-step adherence process in an integrated care post-discharge follow-up program conducted in the hospital and in the primary care settings. Adherence step 1 consisted of successful referral from hospital to municipality, and step 2 was the completion of post-discharge follow-up visits to the patient carried out by general practitioner and municipality nurse (see Figure 1). There was a markedly low degree of adherence in both steps: only 69% of eligible patients were successfully referred from hospital to municipality setting, and barely over half (54%) of the referred patients actually received the post-discharge follow-up visits. Furthermore, we found that levels of adherence were not associated with patients’ physical or social vulnerabilities, such as comorbidity, no spouse or no children living nearby. Hence, our findings do not indicate that the levels of health professional’s adherence to the program guidelines was biased toward more frail patients. In contrast, both steps of adherence were significantly associated with organisational factors, pointing towards potential problems in the vertical integration of the care system [24] that involve hospitals, municipalities and general practitioners. In addition, contact with municipality prior to admission was associated with successful referral, and being registered with a solo practice general practitioner was negatively associated with the odds of receiving post-discharge follow-up visits. The only patient-related factor was gender, as female patients had higher odds of receiving visits than males.

Previous studies have shown that the degree of complexity e.g. engagement of more professional groups and more organisations, tends to impede successful implementation [25]. Managing complexity is critical in the care of older patients with multifaceted health problems, who depend on multiple providers of health care [8]. In the post-discharge follow-up program, although adherence was relatively higher in the hospital/municipality setting (69% referred) than in the primary care setting (54% of post-discharge follow-up visits carried out), adherence levels differed significantly at all organisational levels (Tables 2, 3 and Figure 3), a difference that most likely reflects the complexity of vertical integration in general. Similar studies of post-discharge follow-up programs completed in Denmark, have likewise reported low level of adherence [26]. In Region Zealand, 2014, one regional and six local inter-organizational steering boards, including general practice and municipality as well as hospital managers, monitored the program implementation and discussed the need for tailoring of post-discharge follow-up visits. Yet, an earlier qualitative study of post-discharge follow-up also showed that interdisciplinary cooperation posed challenges to program implementation [27]. Likewise, a qualitative review comparing processes of both interdisciplinary and inter-organisational cooperation concluded that implementation of inter-organisational cooperation faced greater challenges due to factors such as difference in corporate cultures, geographical distance, and formal paths of communication, which should be addressed to achieve better implementation [28]. According to Valentijn et al. [24], organisational integration is complicated by differences in professional roles and culture. Furthermore, organisational integration is dependent on the degree of systemic integration [24]. The significant difference in level of adherence between hospitals and municipalities, possibly reflect more general patterns of inter-organisational cooperation as part of implementation context [27]. In the post-discharge follow-up program, the 889 (54%) patients who received municipal home care prior to index admission (Table 2) had 37% higher odds of successful referral from hospital to municipality. This difference could be a result of technical issues, since in the case of non-registered patients, municipal staff had to search for post-discharge follow-up referrals in parts of the digital system that were not systematically used, with the likelihood of more errors occurring.

Finally, our study identified only one patient-specific factor that was significantly related to the level of adherence: gender. Women were more likely to receive post-discharge follow-up visits compared to men. Since previous studies have shown gender inequity in health care, the issue of gender is not unique to the program described here [29]. Although the data in this study does not allow for further investigation, we nevertheless recommend that gender equity be promoted in integrated care.

### Strengths and limitations

This study is based on unique monitoring data from hospitals and municipalities in a Danish region that was obtained by hospitals and municipalities in 2014. The data provides novel insight to understanding the dynamics of adherence in the post-discharge follow-up program, since we were able to follow the total cohort of patients who were screened and assessed as eligible for the program in Region Zealand. Since, additional Danish studies report low level of adherence for post-discharge follow-up visits, we consider the results to be generalizable, at least to settings with similar health care systems [26,30]. However, we also faced limitations. The adherence concept can be divided into content, coverage, frequency and duration of an intended intervention [2]. This study evaluated only coverage and frequency. Another limitation of this study is the occurrence of missing data as concerns level of education (7% missing values), distance to general practitioner (21% missing values), and type of general practitioner (9% missing values). We used conditional imputation, a technique recommended for dealing with missing values within independent variables [31]. Due to missing values, we chose not to include distance to general practitioner in the multiple regression model (model 2). In addition, we had no data on the total group of patients who were eligible, but were not screened. Municipality staff was responsible for obtaining information on received referral as well as completed visits. There could possibly have been made errors in registration. We did not have data to unravel these potential errors.

## Conclusions

We studied adherence to a new nationwide integrated care program involving health professionals in hospital, municipality and general practitioner settings. We found substantial lack of program adherence in terms of insufficient referral of patients who were clinically eligible for the post-discharge follow-up program (69% adherence at step 1) and in the delivery of the post-discharge follow-up program for those patients who had already been referred from hospital to the municipality (54% adherence at step 2). Starting out from a broad analysis of patient-specific and organisational variables, we found that both steps of adherence were most affected by organisational factors, indicating challenges in the vertical integration of care. Degrees of adherence thus differed significantly at all organisational levels, by hospitals, municipality and general practitioner type. When comparing these results to the existing literature, it appears that lack of adherence is associated with the complexity of implementation that extends across organizational levels of health care. Furthermore, this study revealed gender inequity in adherence at step 2, since men had lower odds of receiving post-discharge follow-up visits than women. We suggest that future studies explore how to strengthen adherence to integrated care activities at the organisational level, and how to prevent gender inequity in integrated health care.
